# Tendency to contact general practice instead of self-care: a population vignette study

**DOI:** 10.3399/bjgpopen20X101024

**Published:** 2020-04-16

**Authors:** Alicia O'Cathain, Rebecca Simpson, Miranda Phillips, Jon M Dickson

**Affiliations:** 1 School of Health and Related Research, University of Sheffield, Sheffield, UK; 2 NatCen Social Research, London, UK; 3 The Academic Unit of Primary Medical Care, The Medical School, University of Sheffield, Sheffield, UK

**Keywords:** general practice, heathcare-seeking behaviour, healthcare utilisation, patients, health surveys

## Abstract

**Background:**

Demand for general practice in the UK is higher than supply. Some patients seek appointments with GPs for minor illnesses rather than self-care.

**Aim:**

To identify the characteristics of people with a tendency to contact GPs rather than self-care.

**Design & setting:**

A national survey of the British adult population was undertaken in 2018, which included vignettes.

**Method:**

Two vignettes focused on illness in adults: half of responders completed a vignette about cough and sore throat for 3 days, and the other half completed a vignette about diarrhoea and vomiting for 2 days. Logistic regression was undertaken to identify characteristics associated with contacting GPs compared with dealing with the problem themselves, calling NHS 111, or contacting another service, including a pharmacist.

**Results:**

The response rate was 42%, with 2906 responders. Responders were twice as likely to select ‘contact GP’ for the diarrhoea and vomiting vignette than for the cough and sore throat vignette (44.7% versus 21.8%). Factors associated with tendency for GP contact included being aged >75 years (odds ratio [OR] 2.0, 95% confidence interval [CI] = 1.2 to 3.2); from black, Asian and minority ethnic (BAME) communities (OR 2.1, 95% CI = 1.5 to 3.0); feeling overwhelmed by unexpected health problems (OR 1.4, 95% CI = 0.99 to 2.1); lower health literacy (OR 1.2, 95% CI = 1.0 to 1.4); and believing that general practice is not overused (OR 1.3, 95% CI = 1.1 to 1.7).

**Conclusion:**

Type of symptom, personal characteristics, and population beliefs about general practice utilisation explain the tendency to contact GPs for minor illness amenable to self-care.

## How this fits in

Clinical workload in general practice is increasing. People contact GPs with minor illnesses that could be addressed using self-care, or advice from NHS 111 or a pharmacist. Addressing low levels of health literacy generally and for specific symptoms may be worth pursuing as interventions to reduce contacts with GPs for minor illnesses.

## Introduction

Clinical workload in primary care in the UK is increasing.^1^ The number of GPs per 100 000 population may be decreasing.^2^ Only 62% of patients seeking a same-day appointment in 2018 received one.^3^ Any mismatch between supply and demand can be addressed by increasing supply of general practice or by reducing demand. It is estimated that millions of general practice contacts annually in the UK are for minor illnesses that could be dealt with at community pharmacies.^4^ These types of contacts with general practice may be labelled ‘inappropriate’.^5^ This term is best avoided because it does not reflect the moral dilemma faced by patients in their help-seeking,^6^ and the challenges faced by patients as they attempt to take responsibility for their health, while not being judged as wasting the time of a service.^7^ Judgments about the necessity of service use are also fraught with difficulty because of concerns that some patients do not contact general practice when they have serious symptoms,^8^ and the fact that judgments regarding legitimate reasons for service use may vary between individual health professionals.^4,9^

There is evidence that people prefer to look after minor ailments themselves or visit a pharmacy,^10,11^ and contact a GP for more serious symptoms.^10,12^ Yet there is still scope for reducing contacts with GPs for minor illnesses.^4^ Identifying the characteristics of people who choose to contact GPs for minor illnesses instead of self-care may help to identify interventions to reduce this type of demand for general practice. People contacting a GP will seek a same-day (urgent) appointment or a booked (routine) appointment. Demand for same-day appointments can be classed as demand for urgent care. As part of a wider study of demand for emergency and urgent care, a population survey was undertaken to identify attitudes to help-seeking when faced with an unexpected health problem that was not life threatening.^13^ Vignettes were used to explore tendency to contact different services for minor illnesses and injuries. In the analysis reported here, the aim was to identify influences on tendency to contact a GP for minor illnesses compared with self-care options.

## Method

### Design

The study design was a cross-sectional population survey that included vignettes.

### Survey sampling

NatCen Social Research conduct an annual survey in Great Britain called the British Social Attitudes Survey to measure the social attitudes of the population.^14^ The survey is designed to yield a representative sample of adults aged ≥18 years. In 2018 NatCen undertook a multi-stage design in three stages. Great Britain is divided geographically into approximately 9000 postcode sectors. First, NatCen selected 395 postcode sectors with probability proportional to the number of addresses in each sector. Second, they selected 26 addresses in each sector to produce 10 270 addresses. Third, interviewers called at each address and listed all those aged ≥18 years before randomly selecting one adult to interview. For practical reasons, the sample was confined to those living in private households; people living in institutions were excluded.

The sample was divided into four parts where each part (of around 1000 responders) was nationally representative in its own right. The University of Sheffield used funding from the National Institute for Health Research to purchase a set of questions in three parts of the survey, that is, a sample size of around 3000. This sample size was chosen because it offered sufficient statistical power for sub-group analyses.

### Mode of administration

The mode of administration was face-to-face computer assisted interviews. Before calling at the address, a letter including an unconditional incentive (a Post Office voucher) was sent to each selected address informing residents that an interviewer would visit. Interviewers then visited and completed most of the questionnaire face-to-face. A small number of questions were asked through a self-completed paper questionnaire collected by the interviewer or posted by the responder. Data collection was undertaken July 2018–November 2018.

### Questionnaire

The 2018 questionnaire covered a range of topics. A 60-item module exploring population views of help-seeking for unexpected health problems that were not life-threatening was purchased. Items were based on: a realist review;^15^ early findings from a qualitative interview study, with three sub-groups of the population who were identified as having a higher tendency to contact emergency and urgent care services when this was not clinically necessary; and a workshop with 13 members of the public where potential questions were presented and discussed in small groups. NatCen undertook two consecutive pilots of draft questionnaires on around 50 members of the public before finalising the questionnaire.

### Primary outcome

Three pairs of vignettes were constructed in conjunction with a GP and an emergency department consultant. Each responder completed one vignette from each pair. The vignettes described situations that people might face, and a set of options they could take. One pair of vignettes focused on illness in adults: imagine you have had a cough and sore throat for 3 days; imagine you have had diarrhoea and vomiting for 2 days. The two clinicians considered it clinically unnecessary to call an ambulance, attend an accident and emergency (A&E) department, or contact a GP for these symptoms. This conclusion was confirmed by the NHS Choices website, a national resource for patients (https://www.nhs.uk), which describes self-care as usually adequate for these symptoms. The following options were offered to responders, ordered by level of urgency: call 999 for an ambulance; go to the A&E department; contact a GP (for telephone advice or an appointment, including GP ‘out-of-hours’ service); go to another NHS service, for example, walk-in centre, minor injury unit, pharmacy, or chemist; call NHS 111; deal with the problem myself; and none of these. Because responders could select more than one option, the study compared responders selecting GP contact as their highest urgency option for the vignettes and compared them with responders selecting dealing with the problem themselves, contacting NHS 111, or contacting another NHS service, including a pharmacist, as one of their highest urgency options.

### Factors tested

Fifty-three factors were tested. These factors were described in three categories for ease of reading (Table 1), although all factors were tested together. There were 12 sociodemographic, socioeconomic, and health items. There were 23 items measuring programme theories potentially explaining clinically unnecessary use of emergency and urgent care, which were derived from a realist review undertaken for the wider study.^15^ These programme theories were supported by theories of health behaviour, including Leventhal *et al*’s common sense model^19^ and Andersen’s healthcare utilisation model.^20^ There were 18 items measuring other issues potentially affecting clinically unnecessary use of emergency and urgent care, which were identified from the wider literature. These issues did not feature strongly enough in the realist review to be identified as programme theories.

**Table 1.** Factors tested in the regression

**Socio** **demographic, socio** **economic** **,** **and health**	**Programme theories from realist review**	**Other issues from literature**
1. Age group 2. Sex 3. Ethnic group 4. Geographical region 5. Household with children aged <5 years 6. Social class (based on the Standard Occupational Classification from the UK Office Of National Statistics) 7. Social deprivation quintiles (measured using Index of Multiple Deprivation^16^) 8. Urban/rural status 9. Personal access to internet 10. Car ownership 11. Presence of limiting long-term illness 12. General health^a^	Uncertainty causes anxiety. Items:1. Worry pain is a sign of something serious.2. Not confident in deciding when to see a doctor.^a^Previous traumatic event. Item:3. Did not see doctor in past when the health problem was serious. Need to get back to normal. Items:4. Seeks help if problem causes sleep loss.5. Seeks help if problem impacts on work. Seeking pain relief. Item:6. Does not take medication to stop pain. Stressful lives cause difficulty coping so low burden services sought. Items:7. Feel overwhelmed when have a health problem.^a^8. Find life stressful.9. Has no one to care for them if ill.^a^10. Difficulty taking time off work to see a GP.11. Ease of travel to an emergency department.12. Opening hours of health services problematic.^a^13. Preference for services with no appointments.^a^14. Desire for services open at times convenient to them.^a^15. Willingness to wait in a waiting room to be seen that day. Follow advice of trusted others. Item:16. Check what to do with family and friends. Perceptions and experiences of services. Items:17. Preference for emergency departments because can get tests done quickly.^a^18. Beliefs about emergency department doctors knowing more than GPs.^a^19. No confidence in their GP.^a^ Frustration with access to GP. Items:20. Ease or difficulty getting GP appointment.21. Whether registered with GP.22. Whether responsibilities make it difficult to see a GP.^a^23. Belief that people use emergency departments because they can’t get a GP appointment.	Awareness of services. Items:1. Confidence in knowing the range of services available.2. Confidence in finding opening times of services.3. Confidence in knowing what tests are available where.4. Confidence in how to contact a GP out of hours.5. Likelihood of looking up a problem on the internet.6. Likelihood of looking up where to go on the internet. Recursivity and/or learnt behaviour.^17^ Item:7. See having tests done as validating attendance at a service.^a^Health literacy, measured using the Health Literacy Questionnaire.^18^ Two of the 9 domains of this instrument were used, each 5 items long. Items:8. Ability to communicate with health professionals.^a^9. Ability to understand health information.^a^Recent use of services for self or others. Items:10. When last used emergency ambulance.11. Frequent use of ambulance.12. When last used emergency department.13. Frequent use of emergency department.14. When last used GP. Perceptions of service overuse. Items:15. Beliefs that too many people use 999 ambulance unnecessarily.16. Too many people use emergency departments.17. Too many people use GP.18. People are reluctant to use emergency departments.

a Variable on self-completed part of questionnaire.

### Analysis

For the vignettes, a binary variable was created based on whether responders chose to contact a GP versus self-care; that is, chose to deal with the problem alone, contact NHS 111 (a 24-hour urgent telephone helpline), or contact another NHS service, including a pharmacist. Options of contacting an emergency ambulance, attending an emergency department, and other options, such as ‘don’t know’, were excluded from the analysis.

IBM SPSS Statistics software (version 25) was used for the logistic regression. First, prior to starting the regression, some categories of some variables were collapsed where numbers were small. Second, a univariate analysis was conducted, testing each of the 53 independent variables. Third, the statistically significant variables from the univariate analysis were tested (where *P*<0.05) in a complete case multivariable logistic regression, using backwards elimination with a cut off of 0.05 for selection. Backwards elimination has advantages over forward selection when variables are correlated.^21^ As the level of missing data was low (apart from the missing values for self-completion questions), missing data were treated as missing and no methods of imputation were used. Fourth, differences by vignettes were tested by testing interaction terms between vignettes completed and each of the factors in the final multivariable model. The results are presented as ORs with 95% CIs.

## Results

### Response rate and non-response bias

The overall survey response was 42%, with 2906 responders completing the module during face-to-face administration. Only 2309 (79.5%) of those interviewed returned the self-completed part of the questionnaire. There was non-response bias. Response was higher where there were no barriers to entry such as locked gates, if the general condition of the address was better than other addresses in the area, and for flats rather than detached houses.^14^

### Description of sample

The characteristics of the full sample are displayed in Table 2. For example, 13.9% of the sample were aged ≥75 years or older and 11.5% were from BAME communities.

**Table 2.** Characteristics of the survey sample, *N* = 2906.

**Variable**	***n* (%**)	**Data missing, *n***
**Age, years**		5
18–24	169 (5.8)
25–34	384 (13.2)	
35–44	467 (16.1)	
45–54	469 (16.2)	
55–64	508 (17.5)	
65–74	499 (17.2)	
≥75	405 (14.0)	
**Sex**		0
Male	1257 (43.3)
Female	1649 (56.7)	
**Ethnic group**		0
White origin	2572 (88.5)
Black Asian and minority ethnic (BAME) communities	334 (11.5)	
**Social class** ^a^		92
I	214 (7.6)
II	1039 (36.9)	
III (non-manual)	569 (20.2)	
III (manual)	416 (14.8)	
IV / V	524 (18.6)	
Armed Forces	52 (1.8)	
**Urban/rural categorisation**		0
Urban	2241 (77.1)
Rural	665 (22.9)	
**Long-term limiting illness**		0
None	1766 (60.8)
Non-limiting	586 (20.2)	
Limiting	541 (18.6)	
Don’t know, refusal	13 (0.4)	
**General health** ^b^		597
Excellent	223 (9.7)
Very good	725 (31.4)	
Good	799 (34.6)	
Fair	360 (15.6)	
Poor	163 (7.1)	
Can’t choose or not answered	39 (1.7)	

^a^Based on the Standard Occupational Classification from the UK Office Of National Statistics. ^b^Self-completed.

### Tendency to contact GP versus self-care

Responses to the vignettes are shown in Table 3. The first two columns show how all 2906 responders completed the vignettes. The second two columns show responses for those included in the logistic regression. Responders were twice as likely to select ‘contact a GP’ for the diarrhoea and vomiting vignette than for the cough and sore throat vignette, and they were more likely to call NHS 111.

**Table 3.** Percentages of sample selecting options for minor illness vignettes.^a^

	All responders,*n* = 1471	All responders,*n* = 1435	Included in logistic regression,*n* = 1395	Included in logistic regression,*n* = 1327	Categorisation of GP contact versus self-care
Cough and sore throat,*n* (%)	Diarrhoea andvomiting,*n* (%)	Cough and sore throat,*n* (%)	Diarrhoea andvomiting,*n* (%)	
Call 999 for an ambulance	4 (0.3)	22 (1.5)	–	–	
Go to the A&E department	17 (1.2)	73 (5.1)	–	–	
Contact a GP (for telephone advice or an appointment, including GP ‘out-of-hours’ service)	320 (21.8)	641 (44.7)	311 (22.3)	615 (46.3)	GP contact
Go to another NHS service, for example, walk-in centre, minor injury unit, pharmacy, or chemist	262 (17.8)	260 (18.1)	256 (18.4)	246 (18.5)	Self-care
Call NHS 111	42 (2.9)	229 (16.0)	37 (2.7)	216 (16.3)	Self-care
Deal with the problem myself	1026 (69.7)	693 (48.3)	1024 (73.4)	681 (51.3)	Self-care
None of these	45 (3.1)	14 (1.0)	–	–	
Refusal or don’t know or missing	10 (0.7)	7 (0.5)	–	–	

a Multiple options could be selected; therefore, percentages add to more than 100.

### Factors explaining tendency to contact a GP

The 926 (34.0%) responders who ticked ‘contact a GP’ were compared with the 1796 (66.0%) who ticked a self-care option (other NHS service including a pharmacist, call NHS 111, deal with it themselves), totalling 2722 responders. Factors with *P*<0.05 in the univariate analysis are displayed in Tables 4–6, for the socio-demographic factors see Table 4, for programme theories see Table 5 and other issues see Table 6. Factors for the complete case analysis of 2148 responders in the final multivariable model are displayed in Tables 4–6.

**Table 4.** Sociodemographic characteristics of tendency to contact GP rather than self-care for minor illness.

Variables	Univariate (*N* = 2722),OR (95% CI), *n*	*P* value	Final multivariable model (*N* = 2148),OR (95% CI), *n*	*P* value
**Age** **, years**	<0.001	<0.001
18–24	1.0 (ref), 158		1.0 (ref), 123	
25–34	1.0 (0.7 to 1.5), 348		1.0 (0.6 to 1.6), 269	
35–44	0.9 (0.6 to 1.4), 437		0.8 (0.5 to 1.3), 327	
45–54	0.8 (0.6 to 1.2), 446		0.9 (0.5 to 1.4), 361	
55–64	1.1 (0.7 to 1.6), 484		1.3 (0.8 to 2.1), 393	
65–74	1.4 (0.96to 2.1), 468		1.4 (0.9 to 2.1), 397	
≥75	2.0 (1.4 to 3.0), 376		2.0 (1.2 to 3.2), 278	
Data missing, *n*	5		—	
**Ethnic** **group**	<0.001	<0.001
White	1.0 (ref), 2439		1.0 (ref), 1967	
BAME	1.7 (1.3 to 2.2), 283		2.1 (1.5 to 3.0), 181	
Data missing, *n*	0		—	
**Region**	0.041		
North England	1.0 (ref), 449			
Midlands	1.1 (0.8 to 1.4), 738			
South England	1.3 (0.99 to 1.6), 897			
London	1.5 (1.1 to 2.0), 262			
Wales	1.4 (0.9 to 2.1), 127			
Scotland	1.4 (1.0 to 2.0), 249			
Data missing, *n*	0			
**Car ownership**	0.004		
≥1	1.0 (ref), 1388			
None	1.5 (1.2 to 1.9), 395			
Not asked^a^	1.1 (0.9 to 1.3), 939			
Data missing, *n*	0			
**Personal access to internet** ^a^	<0.001		
Yes	1.0 (ref), 2393			
No	1.8 (1.5 to 2.3), 329			
Data missing, *n*	0			
**General health**	<0.001		
Excellent	1.0 (ref), 207			
Very good	1.0 (0.7 to 1.3), 695			
Good	1.2 (0.9 to 1.7), 763			
Fair	1.4 (0.96 to 2.0), 339			
Poor	1.8 (1.2 to 2.8), 151			
Can’t choose	2.2 (1.0 to 4.5), 35			
Data missing, *n*	532			
**Long** **-** **term limiting illness**	0.004		
None	1.0 (ref), 1656			
Non-limiting	1.0 (0.8 to 1.3), 556			
Limiting	1.4 (1.1 to 1.7), 503			
Do not know	5.2 (1.0 to 27.0), 7			
Data missing, *n*	0			

^a^One of the four samples used by NatCen did not ask about car ownership. BAME = black, Asian, and minority ethnic. OR = odds ratio.

**Table 5.** Factors measuring programme theories: association with tendency to contact GP rather than self-care for minor illness.

Variables	Univariate (*N* = 2722),OR (95% CI), *n*	*P* value	Final multivariable model(*N* = 2148), OR (95% CI), *n*	*P* value
**Worry pain is a sign of something serious**	<0.001	<0.001
Not likely at all	1.0 (ref), 337		1.0 (ref), 261	
Not very likely	1.3 (0.99 to 1.8), 1087		1.3 (0.9 to 1.8), 873	
Fairly likely	1.9 (1.5 to 2.6), 813		1.8 (1.2 to 2.5), 643	
Very likely	2.9 (2.1 to 4.0), 384		2.1 (1.4 to 3.1), 299	
It depends	1.4 (0.9 to 2.3), 101		1.3 (0.7 to 2.4), 72	
Data missing, *n*	0		—	
**Confident in deciding when to see a doctor or self-care**	0.024		
Very confident	1.0 (ref), 947			
Fairly	1.2 (0.99 to 1.4), 1070			
Not very confident	1.1 (0.7 to 1.9), 79			
Never had problem	1.9 (1.2 to 2.9), 94			
Data missing, *n*	532			
**Affecting sleep**	<0.001		
Will not contact GP	1.0 (ref), 1905			
See doctor only if sleep loss	1.7 (1.4 to 2.1) 586			
See doctor if any loss of function	2.4 (1.8 to 3.2) 231			
Data missing, *n*	0			
**Affecting work** ^a^	<0.001		
Will not contact GP	1.0 (ref), 873			
See doctor only if work loss	1.2 (0.98 to 1.4), 1618			
See doctor if any loss of function	2.3 (1.7 to 3.1), 231			
Data missing, *n*	0			
**Likely to take medication to stop pain**	<0.001		
Very likely	1.0 (ref), 1018			
Fairly	1.0 (0.8 to 1.2), 1188			
Not very	0.7 (0.5 to 0.9), 352			
Not at all	0.6 (0.4 to 0.9), 125			
Depends	1.5 (0.8 to 2.9), 39			
Data missing, *n*	0			
**Feel overwhelmed when have** **health problem**		<0.001		0.020
Strongly disagree	1.0 (ref), 465		1.0 (ref), 462	
Disagree	1.6 (1.2 to 2.1), 829		1.3 (0.99 to 1.7), 821	
Neither	2.3 (1.8 to 3.1), 515		1.7 (1.2 to 2.3), 489	
Strongly agree or agree	2.2 (1.6 to 3.0), 285		1.4 (0.99 to 2.1), 283	
Never had problem	2.0 (1.2 to 3.2), 96		1.7 (1.0 to 2.8), 93	
Data missing, *n*	532		—	
**Can take time off work for GP**	<0.001		
Yes	1.0 (ref), 1024			
Yes but not easy	0.8 (0.6 to 1.1), 255			
No	0.7 (0.5 to 1.2), 115			
Not applicable or missing	1.6 (1.4 to 1.9), 1328			
**Travel to** **e** **mergency** **d** **epartment**	<0.001		
Very difficult	1.0 (ref), 572			
Neither	1.1 (0.8 to 1.5), 243			
Fairly easy	0.8 (0.6 to 0.98), 1099			
Very easy	0.6 (0.5 to 0.7), 808			
Data missing, *n*	0			
**Prefer no appointments**	0.010		
Disagree or strongly disagree	1.0 (ref), 650			
Neither	1.2 (0.9 to 1.5), 765			
Strongly agree or agree	1.4 (1.1 to 1.8), 775			
Data missing, *n*	532			
**Willing to wait in waiting room to be seen**	<0.001		
Disagree or strongly disagree	1.0 (ref), 341			
Neither	1.7 (1.1 to 2.5), 164			
Strongly agree or agree	1.7 (1.3 to 2.2), 2217			
Data missing, *n*	0			
**Check with family and friends for what to do**	<0.001		
Not very likely	1.0 (ref), 507			
Not likely	1.2 (0.96 to 1.6), 695			
Fairly likely	1.4 (1.1 to 1.8), 1005			
Very likely	1.8 (1.4 to 2.3), 515			
Data missing, *n*	0			
**Prefer ED for quick tests**	0.004		
Disagree or strongly disagree	1.0 (ref), 1109			
Neither	1.2 (0.99 to 1.5), 760			
Agree or strongly agree	1.5 (1.2 to 2.0), 321			
Data missing, *n*	532			
**Work or looking after family makes it difficult to see GP**	0.004	0.041
Disagree or strongly disagree	1.0 (ref), 1134		1.0 (ref), 1129	
Neither	1.0 (0.8 to 1.2), 432		0.8 (0.6 to 1.1), 403	
Strongly agree or agree	0.6 (0.5 to 0.8), 388		0.7 (0.5 to 0.9), 386	
Not applicable	1.1 (0.8 to 1.4), 236		0.8 (0.6 to 1.1), 230	
Data missing, *n*	532			

ED = emergency department. OR = odds ratios.

**Table 6.** Factors measuring other issues from literature: association with tendency to contact GP rather than self-care for minor illness.

Variables	Univariate (*N* = 2722),OR (95% CI), *n*	Final multivariable model (*N* = 2148),OR (95% CI), *n*
**Awareness of services**		
Know range of NHS services to use	0.007	
Very confident	1.0 (ref), 1366	
Fairly confident	1.2 (1.0 to 1.4), 1113	
Not confident or not at all	1.5 (1.1 to 2.0), 243	
Data missing, *n*	0	
Can find out when NHS services are open	0.001	
Very confident	1.0 (ref), 1412	
Fairly confident	1.3 (1.1 to 1.5), 1089	
Not confident or not at all	1.6 (1.2 to 2.1), 221	
Data missing, *n*	0	
Can find out what tests are available at NHS services	0.031	
Very confident	1.0 (ref), 913	
Fairly confident	1.2 (1.0 to 1.5), 1224	
Not confident or not at all	1.3 (1.1 to 1.6), 585	
Data missing, *n*	0	
Know how to contact GP out of hours	0.007	
Very confident	1.0 (ref), 1262	
Fairly confident	1.3 (1.1 to 1.6), 1001	
Not very confident	1.4 (1.1 to 1.8), 358	
Not at all	1.2 (0.8 to 1.8), 101	
Data missing, *n*	0	
Will look up on internet to see what to do	0.002	
Very likely	1.0 (ref), 406	
Fairly likely	0.9 (0.7 to 1.2), 708	
Not very likely	1.1 (0.9 to 1.5), 639	
Not at all	1.3 (1.0 to 1.6), 952	
Do not know or never had problem	3.1 (1.1 to 8.2), 17	
Data missing, *n*	0	
Will look up on internet to decide what problem is	0.006	
Very likely	1.0 (ref), 629	
Fairly likely	1.0 (0.8 to 1.3), 756	
Not very likely	1.2 (0.9 to 1.5), 434	
Not at all	1.3 (1.0 to 1.6), 887	
Do not know or never had problem	3.4 (1.3 to 10.3), 16	
Data missing, *n*	0	
**Recursivity**		
If tests are done I was right to contact a service	<0.001	0.003
Disagree or strongly disagree	1.0 (ref), 261	1.0 (ref), 259
Neither	1.9 (1.3 to 2.6), 766	1.5 (1.1 to 2.2), 734
Agree or strongly agree	2.5 (1.8 to 3.5), 1163	1.8 (1.3 to 2.5), 1155
Data missing, *n*	532	—
**Health literacy**		
Lower health literacy compared with high health literacy: understand information	<0.0011.4 (1.2 to 1.6), 2153	0.0131.2 (1.0 to 1.4), 2148
Data missing, *n*	569	—
Lower health literacy compared with high health literacy: ability to communicate with professionals	0.021.2 (1.0 to 1.3), 2153	
Data missing, *n*	569	
Recent use of health care		
Ambulance frequency of use	0.024	
0–3 times in past 12 months	1.0 (ref), 2664	
>3 times in past 12 months	1.8 (1.1 to 3.1), 58	
Data missing, *n*	0	
ED use	0.002	
Never	1.0 (ref), 366	
>12 months	0.6 (0.5 to 0.8), 1409	
6–12 months	0.6 (0.4 to 0.8), 345	
3–<6 months	0.7 (0.5 to 0.99), 212	
<3 months	0.8 (0.6 to 0.99), 390	
Data missing, *n*	0	
ED frequency of use	0.039	
0–3 times in past 12 months	1.0 (ref), 2569	
>3 times in past 12 months	1.4 (1.0 to 2.0), 153	
Data missing, *n*	0	
General practice use	<0.001	0.012
>12 months	1.0 (ref), 424	1.0 (ref), 310
Never	1.5 (0.8 to 2.8), 44	1.5 (0.7 to 3.5), 28
6–12 months	1.5 (1.1 to 2.0), 448	1.5 (1.1 to 2.2), 369
3–<6 months	1.2 (0.9 to 1.6), 491	1.0 (0.7 to 1.4), 386
<3 months	1.7 (1.3 to 2.2), 1315	1.3 (0.99 to 1.8), 1055
Data missing, *n*	0	
**Attitudes towards overuse of health services**		
Too many people use GP when not needed	<0.001	0.042
Agree or strongly disagree	1.0 (ref), 1970	1.0 (ref), 1562
Neither	1.4 (1.2 to 1.7), 523	1.3 (1.1 to 1.7), 412
Disagree or strongly disagree	1.4 (1.1 to 1.9), 229	1.2 (0.8 to 1.7), 174
Data missing, *n*	0	
People are reluctant to use EDs when needed	0.021	
Agree or strongly agree	1.0 (ref), 1263	
Neither	0.9 (0.7 to 1.1), 672	
Disagree or strongly disagree	0.8 (0.6 to 0.9), 787	
Data missing, *n*	0	

ED = emergency department. OR = odds ratio.

Seven of the 12 sociodemographic factors were statistically signficant (Table 4). Surprisingly, deprivation was not associated with a tendency to contact a GP for these minor illnesses in the univariate analysis. In the multivariable analysis, older people and people from BAME communities had a greater tendency to contact a GP.

Thirteen of the 23 programme theory factors were statistically significant in the univariate analysis (see Table 5). Surprisingly, participants’ views of how difficult it was to get an appointment with a GP, and levels of confidence in their GP, were not associated with tendency to contact a GP for these minor illnesses in the univariate analysis. In the multivariable analysis, participants who selected ‘contact a GP’ were more likely to worry that symptoms were serious, feel overwhelmed when faced with an unexpected health problem, and have personal circumstances that made it easy to see a GP. In the sample in the logistic regression, 44.0% (*n* = 1197/2722) worried that pain was a sign of something serious, 13.0% (*n* = 285/2190) felt overwhelmed when faced with a health problem, and 17.7% (*n* = 388/2190) had personal circumstances that made it difficult to see a GP.

Fifteen of the 18 other issues were statistically significant in the univariate analysis (see Table 6). For other issues in the multivariable analysis, participants who selected ‘contact a GP’ were more likely to believe that a service doing tests on them was a sign they were right to seek help (an indicator of recursivity), have lower levels of health literacy, have recently used general practice, and believe that people do not overuse general practice. Responses show that 53.1% (*n* = 1163/2190) of the population felt that if tests were done they were right to contact a service, and 27.6% (*n* = 752/2722) did not believe that general practice was overused.

There were only two statistically significant interactions between the factors in the multivariable analysis and the vignette completed: age and recursivity. This indicates that the results generally are not dependent on the vignette completed.

## Discussion

### Summary

A tendency to contact a GP rather than undertake a self-care option was symptom dependent. Responders were twice as likely to select ‘contact a GP’ for diarrhoea and vomiting than for cough and sore throat. Tendency was also related to personal characteristics, including sociodemographics (older people and people from BAME communities), ability to cope with unexpected illness, and health literacy levels, and finally with beliefs concerning the actions services took for previous problems (recursivity) and about overuse of general practice.

### Strengths and limitations

A wide range of factors were tested, which were based on factors identified in the literature as affecting use of emergency and urgent care for minor ailments and in qualitative interviews with users of ambulance, emergency departments, and same-day general practice. There were three limitations: first, there was non-response bias and the sample did not include people living in residential or nursing homes; therefore, some demographics of older people were not represented. NatCen produced weights to ensure the sample was representative of the British population, but these weights could not be used in the type of analysis that was undertaken. Second, the vignette approach identifies tendency rather than actual use of services and it is possible that people may act differently in practice. Third, the options for vignettes did not include contact with a nurse or other clinician at general practice.

### Comparison with existing literature

The majority of responders in the study selected self-care options for the cough and sore throat vignette. This is supported by evidence that people prefer to look after minor ailments themselves;^10,11^ however, this was not the case for the diarrhoea and vomiting vignette, indicating that behaviour is symptom dependent.

The prevalence of some of the factors tested was similar to those found in previous research. The majority of the responders felt that too many people use general practice when they do not need to (72.4%), compared with 66% in a 2003 survey of a general practice population who felt that people use emergency departments or a GP inappropriately.^22^ Some of the factors that were identified as explaining tendency to use a GP for minor illness were similar to a vignette study in a single UK general practice, where concern that a symptom was a sign of something serious explained help-seeking.^22^

It was found that lower health literacy explained tendency to contact a GP for minor illness and this is supported by a study of factors determining parental reassurance for a child with fever in rural general practice.^23^ Parents who lacked knowledge about complications of fever had higher levels of concern before consulting a doctor and were less likely to be reassured after consultation with a doctor, leading to a recommendation that further education of parents is required.^23^

The present study's findings were supported by theories of help-seeking behaviour and primary research on help-seeking in general practice. People who worry that pain is a sign of something serious are displaying anxiety. Anxiety plays a central role in decision making when managing illness,^19,24^ where patients determine the amount of threat posed by a symptom by thinking about what it might be, how long it should last, what might be causing it, and the potential consequences. Similarly, feeling overwhelmed is a sign of lack of coping and this has been identified as a predisposing factor to healthcare utilisation.^20^ There may be some support for the finding about people from BAME communities in the concept of ‘temporalising’, where people decide to wait for a specified amount of time before seeking help. In research published over 50 years ago, this was particularly associated with Anglo-Saxon Protestant patients but did not significantly influence other ethnic groups.^25^

### Implications for research and practice

There are four possible policy and practice implications of these results. First, people were more likely to consider contacting a GP with some symptoms than others. This has implications for educating the population about specific common minor illnesses and how best to deal with them. For example, educating the population about the expected length of time diarrhoea and vomiting can last without it being a cause for concern may help to reduce tendency to contact a GP. This fits with a recent qualitative study of GPs in the UK that identified patient education about self-management of minor illness as important to cope with increasing workload in general practice.^26^ One way of addressing patient education is GPs discussing expectations around help-seeking for minor illness with patients during consultations for minor illness, and the alternative actions they can take when next faced with similar symptoms. However, GPs in the UK are already concerned about their workload and so have expressed a preference for the government rather than GPs to be responsible for this education.^26^ This means that education might best occur using national campaigns of leaflets or online resources. Attempts have been made to educate people registered with a GP about minor illness using leaflets.^27^ Although this intervention met with some success, the authors concluded that the costs might outweigh the small gains in reduced consultation rates.^27^ That study was published in 2001 and the results are worth reinterpretation in today’s context of increased GP workload and the value of reducing it. It may be worth attempting population-level education about specific symptoms and evaluating the impact of these initiatives. Evaluation could also include assessment of missed serious illnesses given concerns that focusing on reducing GP contacts for minor illnesses might stop some people contacting GPs with serious symptoms.

A second implication of the work is that better attention could be paid to health literacy levels within the population. People with lower health literacy levels had a greater tendency to contact a GP for minor illness. Any leaflets or digital sources of information to educate the population about specific symptoms may not be understood by people with low health literacy. Either information will need to be written for people with low levels of health literacy or interventions will be needed to increase levels of health literacy in the population.

A third implication relates to the finding that some people in the survey felt overwhelmed when faced with an unexpected health problem. Inability to cope with minor illness may be caused by a range of issues such as poverty, stressful work, social isolation, or dealing with chronic illnesses for one’s self or family members. Public health interventions may be needed to address these wider social issues.

A final implication relates to the population sub-groups that had higher tendencies to contact a GP for minor illnesses than others. Interventions could be aimed specifically at older adults and people from BAME communities, although further research or review of existing research is probably needed to better understand why these sub-groups contact GPs.

In conclusion, a range of issues explain tendency to contact GPs for minor illness amenable to self-care. These include personal characteristics, type of symptom, and health literacy levels. Population-level interventions are likely to be needed to address these issues.
